# Urea-Reassembled Soy Lipophilic Protein Nanoparticles for Resveratrol Delivery: Structure, Interfaces, and Digestion

**DOI:** 10.3390/foods14173000

**Published:** 2025-08-27

**Authors:** Mingming Zhong, Yufan Sun, Qayum Abdul, Qiufang Liang, Fan Zhang, Haile Ma, Xiaofeng Ren

**Affiliations:** 1 School of Food and Biological Engineering, Jiangsu University, 301 Xuefu Road, Zhenjiang 212013, China; mingming.zhong@ujs.edu.cn (M.Z.); yufan.sun@ujs.edu.cn (Y.S.); abqyum7@gmail.com (Q.A.); lqf@ujs.edu.cn (Q.L.);; 2 Institute of Food Physical Processing, Jiangsu University, 301 Xuefu Road, Zhenjiang 212013, China; 3 Guangdong Intitute for Drug Control, 766 Shenzhou Road, Guangzhou 510663, China; zhangfan04@163.com

**Keywords:** urea-induced reassembly, soybean lipophilic protein, resveratrol, oil–water interface, in vitro digestion

## Abstract

This study investigated the structure, interfacial properties, and digestibility of resveratrol (Res)-loaded soybean lipophilic protein (LP) nanoparticles using a urea-induced disassembly–reassembly approach. Structural analysis confirmed that LP partially restored its secondary to quaternary structures during dialysis, verifying the reversibility of structural reassembly. Analysis of LP–Res nanoparticles showed that increasing urea concentration ([U]) led to the highest encapsulation efficiency (88.32%) and loading capacity (15.91 μg/mg) at 8 M urea. Meanwhile, characterization of interfacial properties indicated that Res-loaded LP–Res nanoparticles improved interfacial features and foam stability, especially under 8U-Res conditions. Furthermore, dynamic in vitro digestion results demonstrated that 8U-Res nanoparticles exhibited sustained release and the highest digestibility (77.8%). These findings reveal the close relationship between LP structural recovery and interfacial functionality, supporting its application as a nanocarrier in nutritional delivery systems.

## 1. Introduction

A promising strategy to enhance the water dispersibility, stability, and bioavailability of poorly soluble nutraceuticals or pharmaceuticals (e.g., curcumin, resveratrol, and β-carotene) involves developing nanocarriers, particularly those utilizing natural protein-based materials [1,2,3]. Among these, soybean lipophilic protein (LP) has attracted significant attention due to its oleosomal protein–phospholipid (OL-PL) composition, which exhibits strong interface features. LP has abundant hydrophobic domains, making it a promising candidate for encapsulating hydrophobic bioactive compounds [4]. However, conventional encapsulation techniques often bind hydrophobic drugs or nutraceuticals to the surface of protein particles through hydrophobic interactions, resulting in limited loading capacity. Moreover, the encapsulated bioactive compounds remain vulnerable to environmental variations such as temperature, ionic strength, and pH [5,6]. Consequently, there is a growing demand for advanced protein-based nanocarriers that can achieve higher encapsulation efficiency and greater stability, particularly in functional food applications.

Recent advancements in the regulation of non-covalent interactions that maintain protein structure have prompted researchers to explore novel strategies for protein disassembly and reassembly. This methodology aims to create nano-scale protein structures with hollow hydrophobic cores capable of effectively encapsulating hydrophobic nutraceuticals or pharmaceuticals [7,8]. Studies have shown that urea, a well-known chaotropic agent, disrupts non-covalent interactions—particularly hydrogen bonds and hydrophobic interactions [9,10], and is frequently employed as a protein denaturant to induce structural unfolding or dissociation. Importantly, urea at appropriate concentrations is safe for human consumption and has been used therapeutically for treating conditions such as hyponatremia [11]. Additionally, research conducted by ref. [12] and ref. [13] explored the dissociation and reorganization of β-conglycinin subunits (α′, α, and β) in phosphate solutions containing varying urea concentrations (0–8 M). Their results demonstrated the formation of β-conglycinin “core-shell” nanoparticles, a unique structure effective for delivering hydrophobic nutrients or pharmaceuticals. However, limited studies have addressed subsequent physicochemical properties, particularly interfacial characteristics, restricting broader application of these nanoparticles. Based on these findings, we note that the unique structure of LP as a natural protein–phospholipid complex confers excellent interfacial properties, and we hypothesize that urea can similarly promote the unfolding and reassembly of LP, thereby enhancing the encapsulation efficiency of hydrophobic compounds without altering its interfacial properties or further improving the related interfacial stability.

In fact, changes in protein structure, surface hydrophobicity, and size significantly affect their interfacial properties, notably at oil–water and air–water interfaces. For example, the addition of rapeseed oil erucic acid (SA) to whey protein isolate (WPI) promotes protein aggregation and results in a 40% reduction in surface dilatational modulus [14], yet foam stability can be improved. This may be due to aggregates blocking the foam lamellae and interfaces, thereby reducing liquid drainage [15]. Another study highlighted that non-covalent interactions between proteins and phenolic compounds reduced protein surface hydrophobicity, enhancing protein solubility and subsequently increasing foam height and stability [16,17]. These findings underscore the close relationship between structural modifications in proteins and their functional attributes. Moreover, interactions between hydrophobic nutraceuticals or pharmaceuticals and proteins critically influence interfacial properties of the resulting complexes. However, the stability of nanoparticles prepared via the urea-induced disassembly–reassembly strategy at oil–water and air–water interfaces has yet to be verified.

This study aimed to evaluate the encapsulation capacity of resveratrol (Res) by urea-induced LP disassembly–reconstruction. Methodologies such as contact angle measurements and large amplitude oscillatory dilatation (LAOD) were used to determine the influence of this strategy on the interfacial characteristics and foam stability of Res-loaded nanoparticles. Additionally, the digestibility of encapsulated Res was assessed. The outcomes of this research have substantial implications for developing nutraceutical nanocarriers with extensive potential applications in functional foods and pharmaceutical formulations.

## 2. Materials and Methods

### 2.1. Materials

The soybeans used in this study were sourced from Harbin, Heilongjiang, China, and had the following composition on a dry weight basis: 9.8% moisture, 42.1% crude protein, 19.7% fat, and 4.4% ash. Olive oil (Yihai Kerry Golden Dragon Fish Food Group Co., Ltd., Shanghai, China) was used in the experiments. Resveratrol and ethanol (99.9%) were obtained from Jiangsu Real-gen Biotechnology Co., Ltd. (Suzhou, Jiangsu, China). The remaining reagents were all of analytical quality. Every experiment utilized deionized (DI) water.

### 2.2. LP Preparation

Soybean lipophilic protein (LP) was isolated from defatted soybean meal following the method described by ref. [18]. Briefly, defatted soybean meal was heated to achieve a nitrogen solubility index of 75%. After adjusting the pH and ionic strength, LP was separated by centrifugation (Hexi Instrument Equipment Co., Ltd., Changsha, China).

### 2.3. Preparation of Recombinant LP Samples Treated with Urea

Treated with urea, LP was dissolved in deionized (DI) water at 1.0 wt% concentration and stirred magnetically overnight. The dispersion was divided into six aliquots, each mixed with different concentrations of urea ([U]; 0, 2, 4, 6, 8, and 10 M). After stirring for 6 h, urea was removed by dialysis, and residual urea concentration was measured by HPLC [19]. Specific measurement details and results are presented in the supporting information (Tables S2 and S3), confirming residual urea content met food safety standards. The samples were collected, partially freeze-dried (Beijing Zhuoyue United Biotechnology Co., Ltd., Beijing, China), and labeled as 0 U, 2 U, 4 U, 6 U, 8 U, and 10 U.

### 2.4. Characterization of Urea-Induced LP Decomposition and Reorganization Structure

SDS-PAGE analysis was performed according to ref. [20]. LP samples, before and after dialysis, were mixed with β-mercaptoethanol, heated for 2 min, cooled, and loaded onto a polyacrylamide gel (5% stacking gel and 12% separating gel). Electrophoresis was run at 30 mA (stacking gel) and 120 mA (separating gel), with gels visualized using a Chemidoc Touch imaging system.

LP samples were diluted to 0.5 mg/mL in DI water. Fluorescence spectra were measured following ref. [18] using a fluorescence spectrophotometer at an excitation wavelength of 280 nm (slit width = 5.0 nm) and an emission range of 200–400 nm.

FTIR spectroscopy was employed to analyze the secondary structure of LP, as outlined by ref. [21]. Thirty-one scans at 5 cm^−1^ intervals within 4000–400 cm^−1^ were recorded. The secondary structure was analyzed in the amide I region (1700–1600 cm^−1^) using PeakFit version 4V software.

The thermal behavior and degree of denaturation of urea-induced LPs were analyzed using a differential scanning calorimeter (DSC; Yanqu Information Technology Co., Ltd., Hangzhou, China) [22]. The maximum heat flow was used to calculate the peak temperature (T_m_), and the peak area of the thermal curve was used to compute the enthalpy change. Heat the 10 mg sample in the aluminum plate from 20 °C to 170 °C at a rate of 5 °C/min using a nitrogen flow of 50 mL/min. Use an empty sealed disk as a reference.

All experiments were repeated three times to ensure accuracy.

### 2.5. Urea-Induced Reassembled LP Microstructure

Dynamic light scattering (DLS) was performed at 23 °C using a Zetasizer Nano-ZS (Liyou Technology Co., Ltd., Malvern, Taiwan, China). Scanning electron microscopy (SEM) was employed to evaluate nanoparticle morphology. Samples were freeze-dried at −50 °C for 24 h, coated with gold, and viewed under SEM at 15–20 kV with 160× magnification [23]. The experiments were repeated three times. Hydrodynamic diameter and the polydispersity index (PDI) were obtained directly from the DLS 3.0 software by cumulants analysis (intensity weighted values) during size measurements.

### 2.6. Preparation of LP-Resveratrol (LP-Res) Nanoparticles

To make a nanoparticle stock solution (5 mg/mL), Res was dissolved in 75% ethanol. The resulting stock solution was then added dropwise to a solution containing LP at pH 7.0 (1.0 wt%) and urea concentrations ranging from 0 to 10 M (mol/L). The final combination had an LP to Res weight ratio of 9:1 (*w*/*w*). The Res and LP were fully combined and swirled for 10 h. Urea was removed from the solution using dialysis, and the residual amount of urea in the solution was measured (Support Information) [18]. The finished LP-Res nanoparticle slurry was either immediately characterized or lyophilized. The resulting samples were labeled as 0U-Res, 2U-Res, 4U-Res, 6U-Res, 8U-Res, and 10U-Res.

### 2.7. Characterization of Urea-Induced LP-Res Nanoparticles

The encapsulation efficiency (EE%) and loading capacity (LA) of Res in nanoparticles were determined using the method proposed by ref. [18]. In short, 3 mL of ethanol was added to 0.2 mL of different nanoparticle solutions (c = 1.0 wt%). After vortexing the mixture for 60 s, the supernatant (organic phase) was collected and the Res concentration in the supernatant was measured using spectrophotometry at 306 nm. The regression equation was used to determine the concentration of Res: y = 0.126x − 0.023 (R^2^ = 0.999). The experiments were repeated three times in parallel to ensure accuracy. Then, the following formula to calculate EE% and LA was used:

(1) $$EE\left(\%\right)=Res\;in\;\frac{nanoparticles}{total\;Res}\times 100,$$

(2) $$LA\left(\%\right)=Res\;in\;\frac{nanoparticles}{total\;protein}\times 100.$$

A droplet of nanoparticle solution was dropped onto a 230 mesh copper grid for transmission electron microscopy (TEM) observation of the internal microstructure composition. Firstly, 1% (*w*/*v*) phosphotungstic acid was used to dye the nanoparticle samples. Subsequently, the sample was dried overnight at room temperature and TEM images were obtained using a JEM 2100F system (Wanjian Information Technology Co., Ltd., Shanghai, China) operating at 80 kV.

### 2.8. Characterization of the Interface Properties of Nanoparticles

#### 2.8.1. Oil–Water Interface

1-anilinyl-8-naphthalenesulfonate (ANS) was used as a fluorescent probe to evaluate the surface hydrophobicity of LP Res nanoparticle samples [24]. In short, the nanoparticles were diluted to a concentration of 1 mg/mL, and then further diluted to a range of 0.001–0.3 mg/mL. A total of 5 mL of solution was mixed with 80 μL of 5 mM ANS reagent and incubated in the dark for 30 min to complete the reaction. At 484 nm, the fluorescence intensity (FI) was measured while being excited at 365 nm (slit width = 5 nm). The slope of the linear relationship between the concentration of nanoparticles and their maximum fluorescence intensity indicates the hydrophobicity of their surface. The experiments were repeated three times.

Contact angle measurements were conducted using a Biolin Theta Lite optical contact angle meter (Biolin Trading Co., Ltd., Shanghai, China) [25]. Briefly, 15 μL of nanoparticle solution was evenly dropped onto a glass slide and allowed to dry naturally. Then, 10 μL of distilled water droplets was added onto the surface of the dried particles and the contact angle formed between the droplets and the sample was measured. The entire measurement process was carried out at 23 °C.

The emulsifying activity (EAI) and emulsifying stability (ESI) of nanoparticles were evaluated using the method described by ref. [26]. A total of 1.5 mL of olive oil was added dropwise to 15 mL of nanoparticle solution (1 mg/mL) and the mixture was emulsified using a homogenizer (FJ300-SH, Aika Instrument Equipment Co., Ltd., Shanghai, China). Then, the lotion at the bottom of the 25 µL beaker was quickly transferred to 5 mL of 0.1 wt% SDS solution. At 0 and 30 min, the absorbance of the emulsion at 500 nm was measured with an ultraviolet spectrophotometer (A_0_ and A_30_). The experiments were repeated three times in parallel to ensure accuracy. EAI and ESI were calculated using the following formula:

(3) $$EAI\left(\frac{m^{2}}{g}\right)=2\;\times \;2.303\;\times \;\frac{A_{0}\times N}{10,000\theta LC}$$

(4) $$ESI\left(min\right)=\frac{A_{0}}{A_{0}-A_{30}}\;\times \;(T_{30}-T_{0})$$

Among them, N represents a dilution factor of 100 times, θ represents the oil phase fraction, L represents the thickness of the test cup of 1 cm, and C represents the LP concentration expressed in g/mL.

#### 2.8.2. Air–Water Interface

According to the method proposed by ref. [27], surface tension and surface expansion properties were measured. Simply put, a droplet of LP Res nanoparticle sample solution is formed at the needle tip using the hanging drop method. The droplet shape is monitored using a drop tensiometer TECLIS (Xinfang Technology Development Co., Ltd., Shanghai, China) for 180 min and fitted to the Young Laplace equation to determine the surface tension. Throughout the experiment, the droplet continuously expanded and deformed. The deformation amplitude was scanned and recorded at a frequency of 0.05 Hz, with deformation ranging from 5 to 30%.

The scanning results were analyzed by plotting a Lissajous figure to establish the relationship between surface pressure (Π) and deformation (ƒ). The specific calculation formula is as follows:

(5) $$\Pi =\gamma -\gamma_{0}$$

(6) $$ƒ=\frac{A-A_{0}}{A_{0}}$$

where γ_0_ and γ, respectively, represent the surface tension of the interface before and after deformation, and A_0_ and A, respectively, represent the interface area before and after deformation.

### 2.9. AFM Measurement of the Microstructure of Nanoparticle Interface

Preparation of Langmuir–Blodgett Films. Protein–phenol complexes were mixed at a ratio of 0.1:1 (*w*/*w*) and then diluted to a final protein concentration of 0.02 wt%. A 200 μL aliquot of the mixture was applied to the surface of a Langmuir film balance (Biolin Technology Co., Ltd., Shanghai, China) at room temperature to form a Langmuir–Blodgett film for protein-stabilized interfacial films. The sample was injected using an airtight syringe and allowed to equilibrate for 30 min. During this period, surface pressure was continuously monitored using a Wilhelmy platinum plate (circumference of 2 cm, height of 1 cm). The protein layer was moved at a rate of 1 mm/min to a freshly cut mica sheet after a steady surface pressure was achieved, and it was then dried to create a film.

An atomic force microscope (AFM, Zhongpin Technology Co., Ltd., Wuxi, China) was used to analyze the interfacial structure of the Langmuir–Blodgett film [27]. Images were recorded in tapping mode using a 0.40 N/m silicon nitride probe. Every picture was taken using a scan area of 2 × 2 μm^2^ and a lateral scanning frequency of 0.977 Hz. For each nanoparticle sample, it was necessary to scan 2–3 positions of the Langmuir–Blodgett film to ensure the accuracy of the generated AFM images.

### 2.10. Foam Properties of Nanoparticles

Foam stability was assessed using a foaming device (Foamscan, Shanghai, China) with nitrogen sparging to produce foam [28]. In short, 36 mL of 0.1% (*w*/*w*) nanoparticle sample was injected into a glass cylinder with a diameter of 6 cm at a gas flow rate of 6 mL/s to produce foam. A camera was used to track the foam volume decline in order to calculate the foam half-life, and the bubble morphology was recorded in order to assess the size of the bubbles. The experiments were repeated three times.

### 2.11. Digestive Properties

The method was slightly modified based on ref. [29]. Briefly, the composition and in vitro gastric digestion parameters for the simulated gastric fluid (SGF) are summarized in Table S1.

The LP nanoparticle solution was placed in a constant-temperature oscillator at 37 °C (Inheco; Suzhou Jiemei Electronics Co., Ltd., Suzhou, China) to mimic gastric oscillation. The SGF was prepared at a 1.25× concentration to closely simulate physiological gastric secretion conditions. To obtain the appropriate electrolyte concentration, four parts of 1.25× SGF were mixed with one part containing pepsin and CaCl_2_ solution. These two solutions (1.25× SGF and pepsin solution) were added separately to the nanoparticle sample at flow rates of 2.0 mL/min and 0.5 mL/min, respectively. Digestive fluid samples (30 mL) were collected from the bottom of the system every 15 min and passed through a 1-millimeter sieve to simulate gastric emptying. The maximum duration of gastric digestion was 180 min.

Pepsin activity was inhibited by adjusting the pH of the gastric digestive fluid to 7.5 using 0.1 M NaOH, simulating the transition from gastric to intestinal digestion. Simulated intestinal fluid (SIF), prepared at a 1.25× concentration (pH 7.5) containing 150 mM NaCl and 39 mM K_2_HPO_4_, was then added to the gastric digestive mixture at a ratio of 1:1. Additional water, bile extract, trypsin, and CaCl_2_ were added to achieve proper electrolyte composition. After simulating intestinal digestion for 120 min, the Res content in the aqueous phase was determined by centrifugation (9000× *g*, 10 min). The experiments were repeated three times.

### 2.12. Statistical Analysis

All measurements were performed in triplicate, and results are expressed as mean ± standard deviation (n = 3). Differences among treatments were analyzed by one-way ANOVA with Duncan’s multiple range test (*p* < 0.05) using IBM SPSS Statistics 21.0 (IBM, Armonk, NY, USA). Graphs were generated in Origin 2024 (OriginLab, Northampton, MA, USA), and figure assembly/layout was completed in Adobe Illustrator 2024 (Adobe Inc., San Jose, CA, USA).

## 3. Results and Discussion

### 3.1. Urea-Induced Disassembly and Reassembly of LP

**Figure 1:**
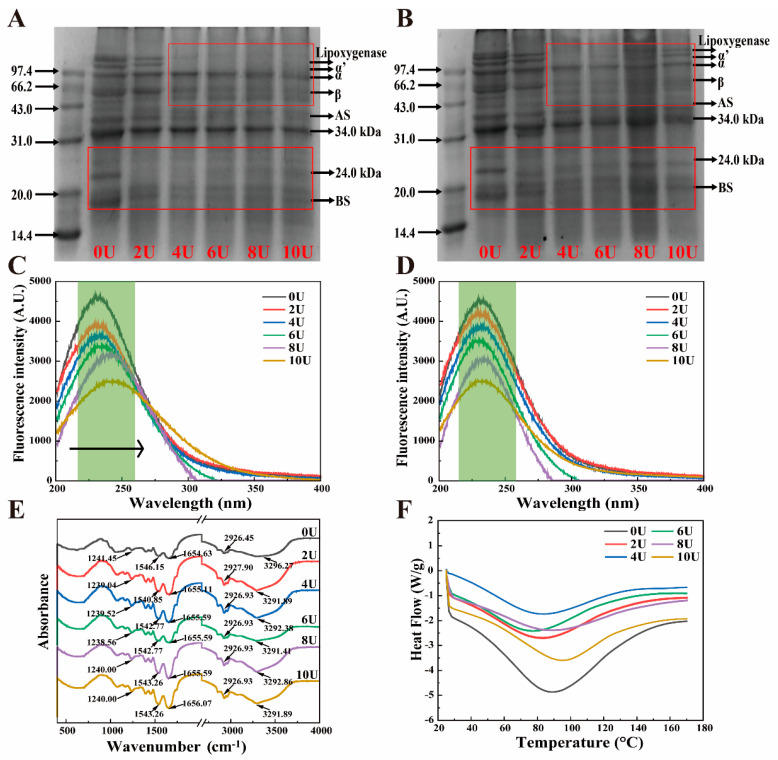
Urea-induced LP disassembly–reassembly structural changes. (**A**,**B**) SDS-PAGE before and after urea induction, (**C**,**D**) fluorescence spectra before and after urea induction, (**E**) FT-IR of reassembled LP, and (**F**) DSC after reassembly.

The SDS-PAGE profiles of urea-induced LP samples, both before and after dialysis, are presented in Figure 1A,B. The primary components of LP include lipophilic proteins such as oleosin (OL, 24 kDa) and lipoxygenase, as well as 7S (α, α′, and β) and 11S (AS and BS) subunits [30]. The pre-dialysis profiles (Figure 1A) show that the interactions stabilizing LP subunits and OL-type structures are predominantly non-covalent, and can be effectively disrupted at urea concentrations above 4 M. At higher [U], LP subunits dissociate, leading to the disappearance of protein bands, which indicates backbone hydrolysis. Following dialysis (Figure 1B), most LP subunit bands are restored as [U] increases, suggesting the involvement of disulfide bonds in LP disassembly and reassembly. The band intensity observed in SDS-PAGE correlates with the sample’s solubility [31]. Notably, the band at 10 M urea, although present, shows the highest intensity, indicating reduced solubility of the reassembled LP, likely due to aggregate formation. A small amount of residual urea may also prevent complete reassembly, contributing to the fading of SDS-PAGE bands (Table S3).

To further investigate the [U]-dependent disassembly–reassembly mechanism of LP, fluorescence spectra were recorded Figure 1C,D. Intrinsic fluorescence emission spectra excited at 280 nm provide insight into the microenvironmental polarity of tryptophan (Trp) and tyrosine (Tyr) residues, offering information about protein conformational changes [32]. Increasing [U] disrupts LP tertiary structure, exposing more Trp and Tyr residues to the polar environment, resulting in a redshift in fluorescence emission. Simultaneously, fluorescence intensity decreases compared to the 0 M control, due to both increased polarity and the quenching effect of urea [12]. After dialysis, LP fluorescence shows a notable blueshift, returning closer to native LP emission wavelengths, indicating partial reversibility of the conformational changes. However, the lower fluorescence intensity compared to untreated LP suggests residual urea remains (Table S3). Combined with SDS-PAGE results, this confirms that the reassembly process maintains LP’s subunit composition. However, at [U] > 8 M, irreversible denaturation and aggregation hinder full structural recovery.

The secondary structural alterations in [U]-induced LP were examined using FT-IR spectroscopy. The amide I band, which predominantly reflects the C=O stretching vibration, is observed in the 1600 cm^−1^–1700 cm^−1^ range, as illustrated in Figure 1E. The region between 1500 and 1600 cm^−1^ is associated with the amide II band, which corresponds to the N–C single bond, N stretching, and N–H bending vibrations. The amide III bands, indicative of N–H deformation, are situated between 1200 cm^−1^ and 1300 cm^−1^ [33]. In this study, [U]-induced LP exhibited broad absorption bands within the 3200–3500 cm^−1^ region, indicating changes in the hydrogen bonding interactions. According to ref. [34], the reassembled LP exhibited a significant blue shift in the hydrogen bonding region (~3290 cm^−1^) compared to untreated LP, indicating modifications in the strength and nature of hydrogen bonds. The valence bonds of N–H and C–O molecules remain relatively stable, while slight offsets were observed in the C–N, CH2, and CH3 bonds. The CH3 groups within the saturated structure were noted between 2850 and 2980 cm^−1^, attributed to the interaction between free and bound –OH and N–H groups (Figure 1E). These results indicate that urea induction significantly alters the original intermolecular and intramolecular hydrogen bonding interactions in LP, likely as a consequence of the reorganization of water adsorption [35]. Additionally, stronger conjugated bonds among the C–N, CH2, and CH3 groups may also contribute to the structural modifications of the urea-modified LP.

Following alteration, the secondary structure of LP exhibited α-helix (1650–1660 cm^−1^), β-sheet (1618–1640 cm^−1^ and 1670–1680 cm^−1^), β-turn (1660–1670 cm^−1^ and 1680–1700 cm^−1^), and random coil structures (1640–1650 cm^−1^) [18]. In comparison to untreated LP, the 8U sample demonstrated a lower percentage of random coils and a higher content of α-helix, indicating improved structural folding, as illustrated in Table 1. This finding suggests that an optimal concentration of urea treatment ([U] < 8 M) facilitates a more ordered reorganization of the LP structure. Conversely, when the urea concentration was increased to 10 M, a decrease in α-helix content and a corresponding increase in random coil content were observed [13]. These alterations suggest that the recombinant LP may become disordered or experience a reduction in structural order at higher urea concentrations.

Changes in the physical state of nanoparticles may be assessed using the peak denaturation temperature in DSC spectra. As shown in Figure 1F, the denaturation temperature of the recombinant LP gradually decreases with increasing urea concentration (0–6 M). This decrease is attributed to the disruption of hydrogen bonding interactions between protein molecules caused by urea, consistent with previous findings [36]. However, with further increases in urea concentration, the denaturation temperatures for the 8U and 10U samples rise, suggesting that the recombinant nanoparticles exhibit enhanced thermal and structural stability [37]. Related studies have shown that these changes come from protein–urea (residual urea after dialysis) interactions that favor the dissociated state to form solvent–solvent interactions that favor the folded state [38]. Overall, the variations in thermal stability are closely dependent on urea concentration and are linked to the distinct structural reorganizations observed in the secondary, tertiary, and quaternary structures of LP as discussed earlier.

### 3.2. Characterization of Urea-Induced LP-Res Nanoparticles

**Figure 2:**
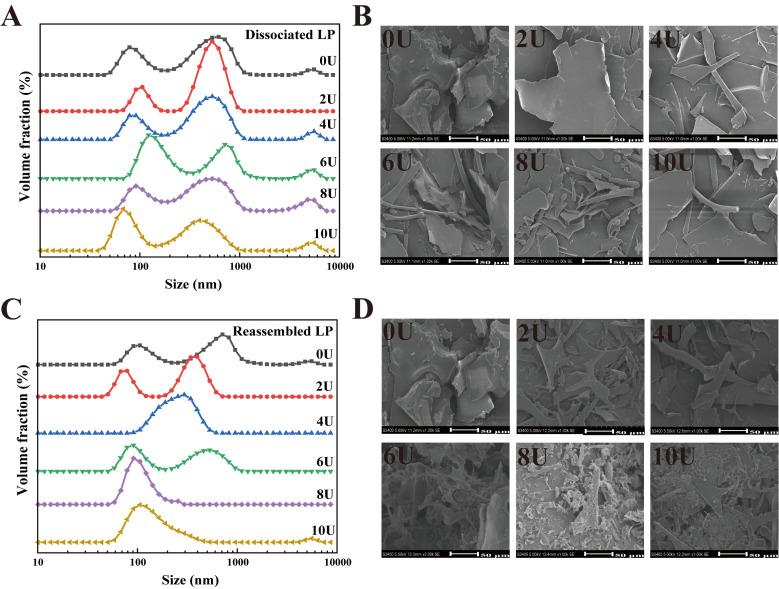
Particle size distribution and microstructure characterization of urea-induced LP disassembly and reassembly. (**A**,**B**) Urea-induced LP disassembly particle size and SEM, (**C**,**D**) LP reassembly particle size and SEM.

The particle size distribution (PSD) of the LP disassembly–reconstruction process at varying urea concentrations (0–10 M) was analyzed using DLS. As shown in Figure 2A, the PSD of untreated LP (0U) exhibited two major peaks centered at approximately 100 nm and 1000 nm, along with a minor peak at around 5 μm. This indicates that native LP undergoes different degrees of aggregation when solubilized in water. Upon the addition of urea, the PSD of urea-treated LP continued to show 2–3 peaks, with an overall increase in aggregation. SEM images (Figure 2B) reveal that as the urea concentration increased, the LP microstructure transformed from flaky or block-like formations to partially rod-shaped structures. Notably, at 8U, a distinct “structural fragmentation” was observed, which may further impact the functional properties of LP [39].

After urea removal by dialysis, LP underwent further self-assembly (Figure 2C,D). From 0U to 6U, the primary PSD peak gradually shifted toward smaller particle sizes, while the peak around 5 μm diminished and eventually disappeared. This suggests a significant reduction in average particle size as urea concentration increased from 0 to 6 M (Table 2). Previous studies have shown that β-conglycinin (β-CG) dissociates completely into its subunits at 6 M urea [7,40]. However, complete dissociation of LP appears to occur at 8U. As shown in Figure 2C, the average particle size was markedly reduced to 208.42 ± 7.46 nm, indicating a transition from bimodal or multimodal to unimodal PSD at 8U. These results suggest that LP molecules—initially composed of mixed subunit types—progressively dissociate into individual subunits as [U] increases from 0 to 8 M. At 10 M urea, the peak around ~100 nm slightly increased, leading to a significant rise in average particle size (*p* < 0.05). SEM images (Figure 2D) further indicate that after urea-induced fragmentation, LP underwent structural reorganization during dialysis. While the LP at 8U displayed reduced particle size, an increase to 10U led to the reappearance of larger sheet-like structures. This phenomenon is likely due to (1) the high content of hydrophobic amino acids in LP’s strongly lipophilic structure, and (2) the higher tendency of the β-subunit of soy protein to denature and aggregate under urea or heat treatment compared to the α- and α′-subunits [41]. Consequently, the increased particle size observed at 10U relative to 8U likely results from the structural unfolding and aggregation of dissociated subunits, particularly those involving the β-subunit. Across the urea series, the particle size pattern reflects a change in assembly mode, from dissociation with size reduction at 0–6 M, to compact reassembly at 8 M, and to aggregation driven by over-unfolding at 10 M.

**Figure 3:**
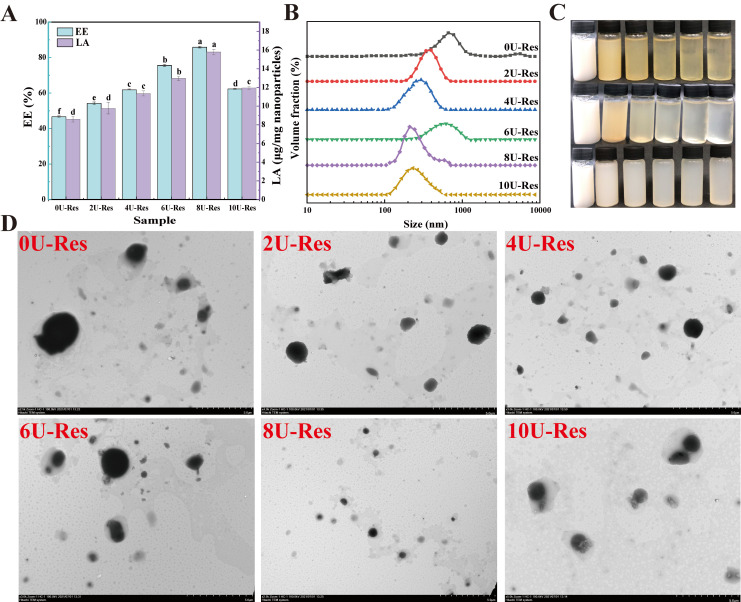
Basic characteristics of LP recombined and coated Res nanoparticles. (**A**) EE and LA, (**B**) particle size distribution, (**C**) solution images, from top to bottom: urea-induced LP disassembly process, recombined and coated Res; from left to right: 0U, 2U, 4U, 6U, 8U, and 10U, (**D**) transmission electron microscopy. Data with different superscripts in the same column indicate significant differences (*p* < 0.05).

The disassembly–reassembly of the secondary, tertiary, and quaternary structures of the nanoparticles, along with changes in their microstructure, can lead to variations in encapsulation properties. Therefore, the effect of urea-induced LP reassembly on the encapsulation of resveratrol (Res) was evaluated through encapsulation efficiency (EE) and loading capacity (LA). As shown in Figure 3A, both EE and LA increased significantly with rising urea concentrations from 0U to 8U, reaching maxima of 88.32% and 15.91 μg/mg, respectively, significantly higher than previously reported results [12]. However, further increasing [U] to 10U led to a decline in EE, which is attributed to nanoparticle aggregation (as depicted in Figure 2). At the same time, this is closely related to the degree of reversible reassembly of the LP structure. The previous analysis showed that treatment with [U] = 8 M can effectively preserve the structural integrity of LP and achieve partial structural reversible reassembly, whereas [U] = 10 M increases LP aggregation, leading to irreversible reassembly or even denaturation. At 8 U the LP subunits likely reassemble with a slightly different relative orientation that makes the hydrophobic core more open and more accessible to the guest. Prior work shows that the spatial arrangement of binding motifs in the host dictates both capacity and binding mode, and that small changes in linker orientation can convert a single pocket into a multi-guest pocket, thereby increasing accessibility and effective affinity [42]. By analogy, transient disruption of intra- and intermolecular interactions by urea followed by dialysis may relax steric hindrance and align key residues at the entry of the LP core, creating a wider or better-oriented pathway for Res to enter and bind. This mechanism agrees with our data: 8 U yields the highest EE and LA together with a unimodal and smaller PSD (DLS, TEM), whereas excessive unfolding and aggregation at 10 U reduce effective accessibility. Environment-driven assembly effects reported elsewhere also support this view, where a change of medium directs the same building blocks to distinct internal architectures [43].

DLS results confirmed that after Res coating, the PSD shifted toward larger sizes, indicating successful encapsulation of Res. These structural changes were also reflected in the nanoparticle morphology (Figure 3C). The LP turned yellowish upon urea treatment, but reverted to a white or colorless appearance following dialysis—an outcome closely related to changes in protein solubility. After Res loading, the nanoparticle suspension appeared milky white with increased turbidity, suggesting particle size enlargement.

The microstructure of Res-loaded nanoparticles (0U-Res to 10U-Res) was further characterized using transmission electron microscopy (TEM). TEM images of natural LP showed individual spherical particles [18]. After Res encapsulation, nanoparticles exhibited a dark hydrophobic core surrounded by a lighter, well-defined hydrophilic outer layer (Figure 3D), confirming the successful embedding of Res within the nanoparticle structure. Particle size first decreased and then increased with rising [U], with most particles appearing loosely aggregated—consistent with DLS findings and further supporting the link between urea-induced structural changes and particle behavior.

Reconstituted LP nanoparticles exhibit two key advantages as carriers for poorly water-soluble bioactives like Res. First, Res crystals can be directly dispersed into 10 M urea solutions containing LP without risk of precipitation. Preliminary tests showed that Res disperses efficiently in 10U, and that urea can be effectively removed by dialysis, with residual urea remaining within safety limits. This is in agreement with findings for β-CG [44]. Second, Res encapsulated using this strategy is not adsorbed onto the protein surface but is embedded within the hydrophobic core of the LP. This mechanism is comparable to curcumin encapsulation within soy protein nanocomplexes, forming core–shell nanoparticles [23,45,46]. Moreover, the ability to control LP disassembly and reassembly by modulating [U] concentration enables precise tuning of the encapsulation process.

### 3.3. Interfacial Properties of Urea-Induced LP-Res Nanoparticles

#### 3.3.1. Oil–Water Interfacial Properties

**Figure 4:**
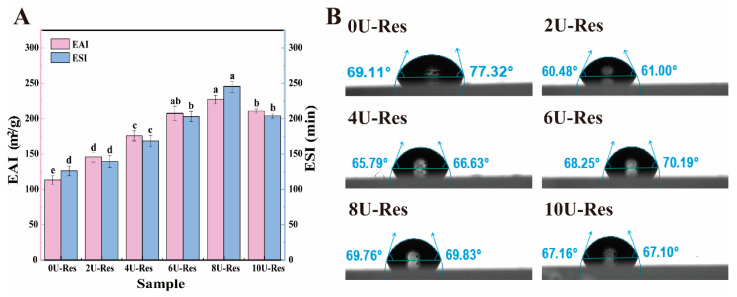
Oil–water interfacial properties of Res nanoparticles coated with LP after reconstitution. (**A**) EAI and ESI, (**B**) contact angle. Data with different superscripts in the same column indicate significant differences (*p* < 0.05).

EAI and ESI were employed to evaluate the emulsifying properties of the nanoparticles, providing insight into their behavior at the oil–water interface (Figure 4A). In line with previous observations, the 8U-Res sample demonstrated the most effective emulsification performance. This result can be attributed to earlier findings indicating that 8U treatment leads to complete unfolding of LP subunits, transforming the molecular conformation from compact sheet-like structures to more flexible and less rigid configurations, such as smaller sheets or rod-like forms. These structural changes enhance nanoparticle flexibility, facilitating better adsorption and rearrangement at the oil–water interface [47]. Furthermore, ref. [48] reported that polyphenol incorporation can significantly enhance the emulsifying capabilities of proteins. Compared to native proteins, partially unfolded or recombined proteins generally exhibit greater molecular flexibility, which contributes to improved emulsification behavior.

To further investigate oil–water interfacial properties, we also evaluated the surface hydrophobicity and contact angle of the nanoparticles (Table 2 and Figure 4B), following the method of [37]. Upon urea treatment, the surface hydrophobicity of LP-encapsulated Res nanoparticles increased significantly. This enhancement is attributed to the disruption of hydrogen bonding within the LP structure, exposing hydrophobic and charged amino acid residues (such as Lys), as illustrated in Figure 1. Previous studies have shown that the increase in hydrophobicity is primarily due to the exposure of buried hydrophobic amino acids on the protein surface [49]. Among all samples, 8U-Res nanoparticles have higher surface hydrophobicity and higher absolute potential, faster adsorption rate, and more compact packing at the interface, resulting in more favorable contact angles and lower dilational moduli; in contrast, excessive expansion and aggregation at 10 M moderately increase the interfacial dilational modulus and reduce surface activity.

In addition, appropriate interfacial wettability is essential for efficient particle adsorption at the interface, enabling the nanoparticles to function as effective emulsifiers. Wettability is typically characterized by the interfacial contact angle. As shown in Figure 4B, the contact angles of urea-treated LP–Res nanoparticles exceeded 60°, which is closely associated with their increased surface hydrophobicity. This observation is consistent with the surface hydrophobicity data and suggests that the nanoparticles exhibit enhanced interfacial activity, promoting the formation of stable oil–water and air–water interfaces [50].

#### 3.3.2. Air–Water Interface Properties of LP-Res Nanoparticles

**Figure 5:**
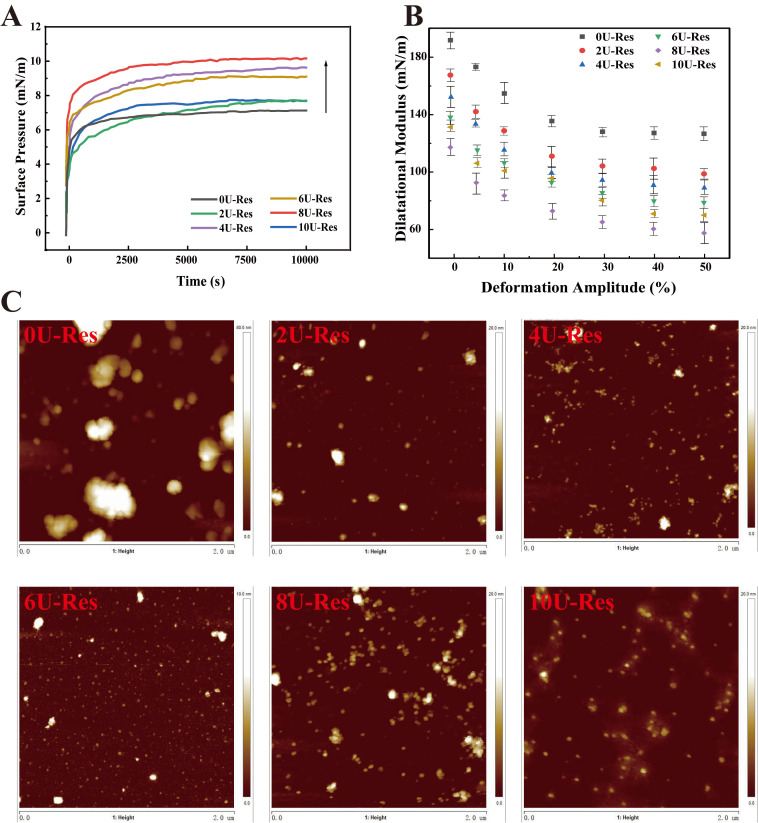
Interface characteristics of encapsulated Res nanoparticles after LP reassembly. (**A**) Interface pressure, (**B**) expansion modulus of elasticity, (**C**) interface AFM.

The surface activity of [U]-induced LP-Res nanoparticles was measured using a drop tensiometer (Figure 5A). The nanoparticles exhibited a rapid increase in surface pressure within the first few seconds, which continued to rise to 6.5–10.3 mN/m after 3 h of adsorption, demonstrating a strong dependence on [U]. The encapsulation of Res by LP under urea treatment led to an overall increase in surface pressure throughout the entire adsorption period, with 8U-Res showing the highest value. This enhancement in surface activity is likely due to the reversible disassembly and reassembly of LP induced by urea, which may facilitate the co-adsorption of both LP and Res at the air–water interface [51].

Figure 5B presents the surface dilatational elastic modulus, measured through amplitude sweep tests across a deformation range of 0–50%, to further characterize the interfacial layer. The elastic modulus gradually decreased with increasing amplitude, indicative of linear viscoelastic behavior dominated by elastic contributions. This trend suggests the formation of a viscoelastic, solid-like interfacial layer [14]. As the amplitude increased, the slope of the surface pressure curve declined, reflecting a progressive loss of interfacial cohesion and an increasing contribution of viscosity to the overall surface response—characteristic of intra-cycle strain softening. Conversely, during compression, intra-cycle strain hardening was observed, as evidenced by surface pressures exceeding those measured during extension. Previous studies attribute this phenomenon to densely packed protein domains at the interface [27,52,53].

**Figure 6:**
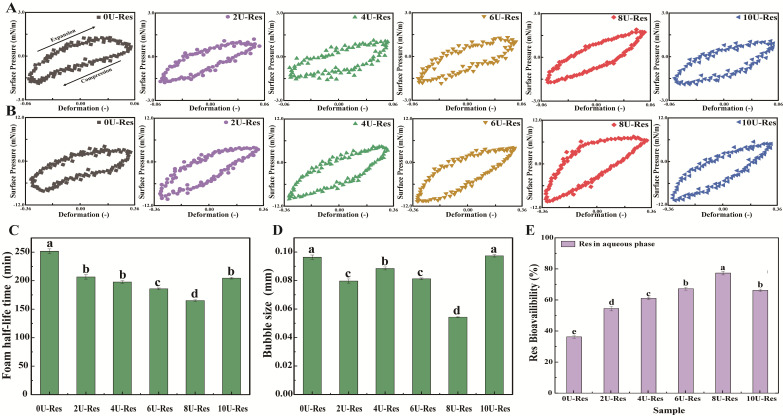
Lissajous plots illustrating the relationship between surface pressure and interfacial deformation of nanoparticle-stabilized air–water films under oscillatory conditions at (**A**) 5% and (**B**) 30% strain amplitudes. Foam stability of reconstituted LP-encapsulated Res nanoparticles: (**C**) foam half-life and (**D**) average foam size. (**E**) In vitro digestibility of Res-loaded nanoparticles.Data with different superscripts in the same column indicate significant differences (*p* < 0.05).

To assess the microstructure of the interfacial film, Langmuir–Blodgett deposition was used to transfer the nanoparticle-stabilized film onto a solid substrate for atomic force microscopy (AFM) analysis (Figure 5C). AFM results revealed that the 0U-Res sample exhibited significant large-scale aggregation, forming a non-uniform interfacial film characterized by thick, densely packed regions—likely composed of aggregated protein [53,54]. With increasing [U], the interfacial films became more homogeneous and uniform, exhibiting continuous, solid-like viscoelastic features. Previous research has shown that such structures strongly adsorb at the air–water interface and contribute to enhanced interfacial stability [14]. To further assess the mechanical behavior of LP-Res interfacial films, interfacial expansion–compression deformation tests were conducted to generate Lissajous curves based on interfacial stress and modulus (Figure 6A,B). This approach provides a valuable tool for probing nonlinear interfacial rheology [55,56].

Figure 6A,B displays the Lissajous plots for all LP-Res nanoparticles under 5% and 30% deformation. The plots are arranged clockwise, with the upper and lower halves representing stretching and compression phases, respectively. The shape of the plots reflects the interfacial characteristics: linear, purely elastic responses produce straight lines, whereas fully viscous responses form circular loops. At 5% strain (Figure 6A), 2U-Res and 4U-Res samples show reduced slope values, indicating lower interfacial stiffness and a softer interfacial network [57]. In contrast, 8U-Res exhibited the steepest slope, reflecting a more rigid interface. Moreover, the Lissajous plot of 8U-Res began to display asymmetric and non-elliptical shapes, indicative of pronounced nonlinear viscoelastic behavior and enhanced interfacial complexity. These nonlinearities became even more evident at the higher deformation of 30% (Figure 6B), confirming the viscoelastic strengthening of the interface at 8U-Res, which may contribute to its superior emulsification properties.

At 30% strain, all LP-Res interfacial systems (Figure 6B) exhibited increased slope and asymmetry in their Lissajous curves, revealing distinct interfacial behaviors under both tensile and compressive stress. Notably, for 8U-Res, the curve showed a marked increase in slope at the onset of the extension phase, indicating a sharp rise in surface pressure, followed by a decrease in slope that approached a horizontal profile toward the end of the stretching cycle. This pattern suggests interfacial strain softening during extension. During the compression phase, surface pressure dropped sharply from 6 mN/m to 10 mN/m, revealing compressive strain hardening at the interface.

These interfacial behaviors can be attributed to dynamic changes in nanoparticle concentration at the interface during high-strain cycles, leading to the formation of a soft, solid-like interfacial film. This underscores the interfacial adaptability and adsorption flexibility of LP-Res nanoparticles. A similar tensile–compressive response was previously observed by ref. [14] in their study of whey protein–rapeseed oil body mixtures at the air–water interface. Overall, all LP-Res nanoparticle systems were capable of forming viscoelastic, solid-like interfacial layers with varying degrees of strength. Among them, 8U-Res established the most robust air–water interface, suggesting enhanced interfacial stability and a strong potential for improving emulsification performance.

### 3.4. Foam Stability of LP-Res Nanoparticles

The average foam size and foam half-life (i.e., the time required for half of the foam volume to collapse) were evaluated by introducing N_2_ gas into the nanoparticle solution to generate foam, thereby assessing both the foaming ability and stability of the nanoparticles. The relevant data are presented in Figure 6C. Previous studies have reported that LPs possess high foam stability and are effective foaming agents [58]. In the present study, the 0U-Res formulation exhibited a high foam half-life of 250.3 ± 13.5 min. However, for the newly formed nanoparticles generated via [U]-induced recombinant LP encapsulating Res, the foam half-life decreased to a range of 168.4–170.2 min. This reduction in foam stability is likely attributed to the lower air–water interfacial dilatational modulus (Figure 5), as ref. [59] demonstrated that the mechanical strength of the interfacial layer surrounding the bubbles is closely related to foam stability.

Interestingly, foam stability was found to be associated with the decrease in interfacial dilatational modulus of nanoparticles formed after [U] treatment (Figure 5) and the reduction in average bubble size (Figure 6D). The 8U-Res sample exhibited the lowest foam half-life, as well as the lowest interfacial dilatational modulus and average bubble size. As demonstrated in reference [59], a decrease in interfacial dilatational modulus leads to a reduction in the mechanical strength of the interfacial layer surrounding bubbles, thereby affecting foam stability. Notably, among all samples, the foam half-life of 10U-Res was slightly longer than that of nanoparticles with [U] ≤ 8 μM. This may be attributed to its larger bubble size, as the formation of aggregated particle structures has been shown to improve foam stability, as evidenced by studies on casein micelles and β-lactoglobulin aggregates [60].

Analysis of Res encapsulation revealed that as the amount of encapsulated Res increased ([U] = 2–8 M), the foam half-life decreased in conjunction with a significant reduction in foam size (*p* < 0.05). Previous studies have shown that the addition of phenolic compounds to proteins can enhance their foaming properties. This enhancement is due to the co-adsorption of phenols and proteins at the interface, which alters protein conformation and increases surface hydrophobicity [61,62]. The nature of phenol–protein interactions plays a critical role in foam stability, as protein aggregates can reinforce foam structures by promoting the assembly of flakes and interfacial particles within the foam matrix [61,63]. Moreover, foam stability can also be enhanced through reduced surface hydrophobicity, improved protein solubility, and increased air–water interfacial stability [62,64]. Collectively, these findings suggest that the foaming behavior of the nanoparticles is predominantly governed by their air–water interfacial properties.

### 3.5. Digestive Characteristics of LP-Res Nanoparticles

Due to the different degrees of reversible reassembly of the LP structure induced by urea, almost all resveratrol molecules can be efficiently encapsulated within the LP molecular structure rather than on its surface. Therefore, it is particularly important to evaluate the digestibility of Res in this delivery system. In vitro dynamic gastrointestinal digestion models have been demonstrated to offer greater efficiency and accuracy than static models when assessing the digestibility of encapsulated compounds, as supported by our previous findings [29], as shown in Figure 6E. The amount of Res transferred into the aqueous phase during digestion serves as a direct measure of its digestibility. It is noteworthy that the digestibility of resveratrol encapsulated by natural LP is significantly low, only 37.98%, because the protease-binding sites of natural LP are buried within the molecular structure, reducing binding efficiency, which is consistent with previous reports. In contrast, [U]-induced reconstituted LP exposes protease-binding sites to varying degrees on the molecular surface, thereby improving the bioavailability of resveratrol in the nanoparticles, reaching approximately 55–78%, and the improvement is statistically significant (*p* < 0.05). Among all formulations tested, the 8U-Res nanoparticles exhibited the highest digestibility rate at 77.8%, significantly higher than the release rate of Res encapsulated using bovine serum albumin-caffeic acid conjugate (62.7%) [65]. The enhanced digestibility can be attributed to the following: (1) the [U]-induced varying degrees of reversible reassembly of the LP structure, enabling the effective encapsulation of Res within the nanoparticle structure; and (2) the excellent interfacial stability exhibited by the LP–Res structure in earlier characterizations. As the digestion process inherently involves dynamic interfacial activity, the improvement in interfacial adsorption and stability facilitates higher digestive efficiency. A stable gastric environment helps deliver a greater proportion of Res to the intestine, thereby enhancing its overall digestibility and bioavailability.

## 4. Conclusions

This study investigated the structural characteristics, interfacial properties, and digestive dynamics of nanoparticles synthesized via the successful encapsulation of resveratrol (Res) using LP self-assembly, facilitated by hydrogen bonding interactions induced through urea treatment. The results demonstrated that hydrogen bonding played a pivotal role in the disassembly and subsequent reorganization of the LP structure. An optimal urea concentration ([U] < 8 M) promoted a more “ordered” LP conformation, whereas higher concentrations led to protein aggregation or denaturation. The “ordered” conformational reassembly of LP significantly increased the encapsulation of Res (EE = 88.32%) while simultaneously improving the emulsifying properties and surface hydrophobicity of the nanoparticles. Interfacial parameters, including surface pressure and foam stability, were highly dependent on urea concentration, with the highest surface pressure observed in the 8U-Res sample. The interfacial film exhibited enhanced structural uniformity and behaved as a homogeneous viscoelastic solid, thereby improving adsorption at the air–water interface and enhancing interfacial stability. Although the foam half-life was reduced, the foam bubble size was also significantly decreased. Importantly, the formation of nanoparticles induced by urea significantly enhanced the bioavailability of resveratrol, reaching approximately 55–78%.

In conclusion, the urea-induced LP Res nanoparticles are suitable nanocarriers for hydrophobic bioactives in oil-in-water foods such as functional beverages, plant-based dairy alternatives, and dressings, and the lyophilized powders can be used as dry premixes for on-demand dispersion. For foam-rich formats, additional stabilizers may be required. For industrial implementation, the laboratory dialysis step can be replaced by food-grade ultrafiltration or diafiltration operated in closed loop and integrated into standard mixing, concentration, and drying lines, with routine quality control specifications for residual urea, particle size distribution and polydispersity, and encapsulation efficiency, together with matrix-specific shelf life and sensory validation.

## Figures and Tables

**Table 1:** Secondary structure content of FT-IR of recombinant LP.

Sample	α-Helix (%)	β-Sheet (%)	β-Turn (%)	Random Coil (%)
0U	30.44 ± 0.1 ^a^	39.45 ± 0.2 ^a^	17.64 ± 0.1 ^e^	12.47 ± 0.1 ^b^
2U	27.69 ± 0.1 ^c^	35.28 ± 0.2 ^c^	22.84 ± 0.1 ^b^	11.36 ± 0.1 ^c^
4U	27.25 ± 0.1 ^d^	34.26 ± 0.1 ^d^	21.73 ± 0.1 ^c^	10.48 ± 0.1 ^d^
6U	27.14 ± 0.2 ^d^	35.20 ± 0.1 ^c^	20.20 ± 0.2 ^d^	12.77 ± 0.2 ^b^
8U	28.42 ± 0.1 ^b^	37.97 ± 0.1 ^b^	21.97 ± 0.2 ^c^	11.35 ± 0.1 ^c^
10U	26.26 ± 0.1 ^e^	33.18 ± 0.2 ^e^	23.85 ± 0.2 ^a^	17.61 ± 0.1 ^a^

Values are mean ± SD (n = 3). Different superscripts within a column indicate significant differences (one-way ANOVA, Duncan’s test, *p* < 0.05). FT-IR secondary-structure fractions were obtained by deconvolution of the amide I band. 0U–10U denote urea concentrations (0–10 M).

**Table 2:** Average particle size, PDI, Zeta potential, and surface hydrophobicity after LP recombination.

Sample	Average Particle Size (nm)	PDI	ζ-Potential (mV)	Surface Hydrophobicity (H_0_)
0U	397.44 ± 10.31 ^a^	0.45 ± 0.23 ^a^	−17.35 ± 0.75 ^e^	2376.63 ± 10.24 ^i^
2U	373.69 ± 5.17 ^c^	0.28 ± 0.25 ^c^	−22.86 ± 0.65 ^b^	3526.74 ± 4.48 ^b^
4U	282.25 ± 6.58 ^d^	0.26 ± 0.13 ^d^	−23.83 ± 0.78 ^c^	4054.54 ± 7.63 ^a^
6U	263.14 ± 7.35 ^d^	0.20 ± 0.15 ^c^	−24.54 ± 0.26 ^d^	4694.31 ± 4.65 ^f^
8U	208.42 ± 7.46 ^b^	0.97 ± 0.14 ^b^	−27.67 ± 0.97 ^c^	5123.55 ± 5.17 ^d^
10U	326.26 ± 3.56 ^e^	0.18 ± 0.25 ^e^	−23.85 ± 0.37 ^a^	4224.66 ± 6.68 ^c^

Data with different superscripts in the same column indicate significant differences (*p* < 0.05).

## Data Availability

The original contributions presented in this study are included in the article/Supplementary Materials. Further inquiries can be directed to the corresponding author.

## Supplementary Materials

The following supporting information can be downloaded at https://www.mdpi.com/article/10.3390/foods14173000/s1, Table S1. Simulated gastric digestive juice composition. Table S2. Mobile phase gradients [solvent A: 20 mM sodium acetate (pH 7.2); solvent B: acetonitrile]. Table S3. Urea remaining after dialysis of recombinant LP.

## Abbreviations

Acronym	Full Term
LP	Lipophilic protein
Res	Resveratrol
EE	Encapsulation efficiency
LA	Loading ability
PSD	Particle size distribution
DLS	Dynamic light scattering
PDI	Polydispersity index
ζ-potential	Zeta potential
H0	Surface hydrophobicity index
FI	Fluorescence intensity
SEM	Scanning electron microscopy
TEM	Transmission electron microscopy
FTIR	Fourier transform infrared spectroscopy
DSC	Differential scanning calorimetry
EAI	Emulsifying activity index
ESI	Emulsifying stability index
ANS	1-anilinyl-8-naphthalenesulfonate
AFM	Atomic force microscopy
SGF	Simulated gastric fluid
SIF	Simulated intestinal fluid
LAOD	Large-amplitude oscillatory dilatation
HPLC	High-performance liquid chromatography
