# Supplementary material for: Urea-Reassembled Soy Lipophilic Protein Nanoparticles for Resveratrol Delivery: Structure, Interfaces, and Digestion
Source: Foods. 2025 Aug 27;14(17):3000. doi: 10.3390/foods14173000 (PMC12428604; doi:10.3390/foods14173000)
Supplement: Supplementary file 1 [file foods-14-03000-s001.zip › foods-3763022-supplementary.pdf]

Table S1. Simulated gastric digestive juice composition.

Ingredient	SGF
Inorganic solution	15.7 mL of 175.3 g/L $NaCl$<br>3.0 mL of 88.8 g/L NaH_2PO_4<br>9.2 mL of 89.6 g/L KCl<br>18 mL of 22.2 g/L $CaCl_2 \bullet 2H_2O$<br>10 mL of 30.6 g/L NH_4Cl<br>6.5 mL of 37% HCl
Organic solution	10 mL of 65 g/L glucose
Digestive enzyme	250 mg/100 mL pepsin

Determination of urea content by HPLC

Urea was determined using a 1200 series HPLC system with solvent degasser system, quaternary pump, autosampler and fluorescence detector (Agilent Technologies, Blackburn, Australia). An Eclipse XBD RP-18 column (150 mm × 4.6 mm I.D., 5 μm) from Agilent Technologies was used for all separations at a constant temperature of 35 °C and the gradients shown in Table S2. The derivatised analyte was detected with excitation and emission wavelengths of 213 nm and 308 nm, respectively, using slit widths of 20 nm.

Table S2. Mobile phase gradients [solvent A: 20 mM sodium acetate (pH 7.2); solvent B: acetonitrile].

Gradient	Time (min)	Solvent (%B)	Flow rate (mL min^{-1})
	0	20	0.45
	0.06	20	0.45
	12.6	50	0.45
1	13.6	100	0.45
	20.6	100	0.8
	22.6	20	0.45
	23.6	20	0.45
	0	20	1.0
	0.06	20	1.0
	12.6	50	1.0
2	13.6	100	1.0
	20.6	100	1.0
	22.6	20	1.0
	23.6	20	1.0
	0	50	1.0
	3.3	50	1.0
	4.0	100	1.0
3	7.0	100	1.0
	7.5	20	1.0
	10.0	20	1.0
	10.5	50	1.0
	12.0	50	1.0

Table S3. Urea remaining after dialysis of recombinant LP.

	Sample	Urea (μg/mL)
	0U	0
	2U	0.08 ± 0.01[d]
Reassembled LP	4U	0.14 ± 0.01[c]
	6U	0.17 ± 0.02[c]
	8U	0.24 ± 0.02[b]
	10U	0.36 ± 0.04[a]

*Values with different superscripts in the same column are significantly different at $p < 0.05$.
